# Impact of sodium alginate energy gel on the marathon performance of amateur runners: a randomized controlled study

**DOI:** 10.3389/fphys.2026.1746392

**Published:** 2026-02-11

**Authors:** Peng Zhang, Zhongke Gu, Kai Xu, Yanrong Zhao, Gangrui Chen, Jiansong Dai

**Affiliations:** 1 Department of Sport and Health Science, Nanjing Sport Institute, Nanjing, China; 2 Global R&D Innovation Center, Inner Mongolia Mengniu Dairy (Group) Co. Ltd., Shanghai M-Action Health Technology Co., Ltd., Shanghai, China; 3 Sports Science Research Institute, Nanjing Sport Institute, Nanjing, China

**Keywords:** continuous glucose monitoring, energy gel, exercise performance, marathon exercise, sodium alginate

## Abstract

**Objective:**

Sodium alginate hydrogel can encapsulate high-sugar substances, alleviate gastric discomfort, accelerate gastric emptying speed, and gradually expand in the small intestine while slowly releasing carbohydrates. This study aimed to evaluate the impact of sodium alginate energy gels versus traditional energy gels on glycemic stability and running performance in marathoners.

**Methods:**

Eighty-one amateur runners were selected and randomly assigned to four groups: elite sodium alginate energy gel (ES), elite traditional energy gel (ET), advanced sodium alginate energy gel (AS), and advanced traditional energy gel (AT). All participants utilized CGM devices for 1 week prior to the race to establish baseline glucose levels. During the marathon, carbohydrates were administered at a rate of 60 g per hour. Individual pace and glucose data were normalized to account for variations among runners of differing skill levels.

**Results:**

The completion times for both runner groups at the same level showed no notable differences. In the AS group, blood glucose fluctuation range during the 11–20 km and 31–40 km segments were found to be lower compared to the AT group. Additionally, the ES group exhibited a significantly smaller percentage change in speed over the 1–42 km segment compared to the ET group, while the AS group also demonstrated a notably lower percentage change in speed for the 1–20 km segment relative to the AT group.

**Conclusion:**

While sodium alginate energy gel did not improve average race pace or final competition results, it significantly enhanced blood glucose stability for advanced runners in specific race segments. Additionally, it improved pace stability for elite runners throughout the race and for advanced runners during the first half. These findings suggest that sodium alginate energy gels may have potential benefits in promoting metabolic regulation during endurance exercise.

## 1 Introduction

With the increasing popularity of marathon running in the country, more amateur sports enthusiasts are eager to participate in these events. Runners of various skill levels are exploring methods to enhance their performance through improved training and nutrition. Marathon running is generally characterized as a prolonged, high-intensity endurance challenge (Saris et al., 2003). Studies have shown that carbohydrate supplementation during endurance activities can help sustain or boost athletic performance (Coyle et al., 1983; Coyle et al., 1986; Coggan and Coyle, 1987). Consuming carbohydrates may enhance long-term endurance, likely due to the maintenance of muscle glycogen and blood glucose levels (Coyle et al., 1986; Tsintzas and Williams, 1998). This notion has been supported by research utilizing continuous glucose monitoring (CGM), which revealed that during ultramarathons, a drop in blood glucose correlates with a decrease in running speed (Sengoku et al., 2015; Ishihara et al., 2020), and greater fluctuations in blood glucose can also hinder running speed (Ishihara et al., 2020). Current recommendations suggest that athletes should maintain a high carbohydrate intake during intense exercise exceeding 2.5 h, with a suggested hourly intake of up to 90 g (Thomas et al., 2016). However, in marathon races, runners’ energy demands and blood glucose levels undergo significant changes as the race progresses. Thus, an effective energy supplementation strategy for marathons must consider these variations to determine the optimal timing and types of supplements to support or improve athletic performance.

Energy gels, a common carbohydrate supplement in endurance sports, are favored in high-intensity events like marathons due to their convenience and quick energy delivery (Baur and Saunders, 2021; Coyle, 1992). Traditional energy gels help maintain blood glucose levels and postpone fatigue by quickly supplying exogenous glucose (Coyle, 1992). Nonetheless, these gels have notable drawbacks regarding osmotic pressure control, gastrointestinal tolerance, and glycemic instability, especially in the demanding conditions of marathons. The osmotic pressure of many commercial energy gels is typically much higher than that of human plasma, which can slow gastric emptying and result in bloating and nausea (Zhang et al., 2015). While hypertonic gels can deliver energy quickly, they may cause fluctuations in blood sugar, negatively impacting endurance performance. Conversely, isotonic gels theoretically promote faster gastric emptying but often contain lower carbohydrate concentrations per serving (Zhang et al., 2015), making it challenging to meet the energy needs of prolonged high-intensity exercise, thus creating a dilemma for athletes trying to balance energy efficiency and gastrointestinal comfort. Therefore, despite their benefits in providing immediate energy, traditional energy gels require significant improvements in taste, osmolality management, blood sugar stability, and gastrointestinal tolerance through innovative sustained-release carbohydrate systems and formula enhancements to ensure a rapid, enduring, and stable energy supply while improving the overall user experience.

Currently, energy gel technology is advancing, including the development of sodium alginate hydrogel technology (Sutehall et al., 2018). This method encapsulates carbohydrates in alginate-based hydrogels, allowing for high-concentration carbohydrate intake while improving gastric emptying and absorption rates. Alginate, a high molecular weight polymer derived from seaweed (Nielsen et al., 2024), has previously been used in protein encapsulation for drug delivery (Tønnesen and Karlsen, 2002). Earlier research on alginate-based energy supplements involved beverages combining alginate and pectin. The proposed mechanism is that when this mixture encounters the acidic environment of the stomach, it forms a pH-sensitive hydrogel that encapsulates the carbohydrates. This gel then transitions to the small intestine, where it gradually breaks down and releases carbohydrates for absorption as the intestinal pH rises (Sutehall et al., 2020; Sutehall et al., 2022b; Rowe et al., 2022). Previous findings indicated that sodium alginate hydrogel can enhance the initial gastric emptying rate of high-concentration carbohydrate beverages at rest (Sutehall et al., 2022a), but endurance exercise performance and pacing outcomes have not yet been systematically investigated, leaving uncertainty as to whether these physiological advantages translate into meaningful, real-world benefits during actual marathon competition. In contrast to previous studies conducted in controlled environments (Baur et al., 2019; Sutehall et al., 2020; Rowe et al., 2022), this research utilized carbohydrates that were pre-encapsulated in sodium alginate hydrogel prior to ingestion, and it was carried out in the context of an actual marathon race. The primary focus was on the practical application of the new sodium alginate energy gel during marathon events. This study aims to determine whether the energy gel formulated with sodium alginate hydrogel technology can more effectively enhance the exercise performance of amateur runners in marathon races compared to traditional energy gels. Furthermore, this study employed continuous glucose monitoring (CGM) devices to track the blood glucose levels of runners (Blake et al., 2024), examining the differences in the impact on blood glucose indicators between the sodium alginate-based energy gels and traditional energy gels.

## 2 Participants and methods

### 2.1 Participants

The sample size was estimated using G*Power software, implementing a repeated measures design. This calculation was based on the effect size reported in previous studies on carbohydrate hydrogel beverages, with the effect size set at 0.25 (Baur et al., 2019), a P-value of 0.05, and a power of 0.8 (Sutehall et al., 2020). Following this calculation, the total sample size was determined to be 68 participants, with 17 individuals in each group. To account for potential sample attrition, the planned number of recruits has been increased to approximately 23 participants per group. This study selected participants including advanced runners and elite runners, all of whom were male. Elite participants were required to have completed a full marathon in under 3 h and 25 min within the last 3 years, while advanced runners needed a marathon completion time of less than 4 h and 30 min. A total of 93 individuals were recruited through voluntary sign-ups, screening, and review, which included assessments of age, height, weight, past personal best (PB) times, and running frequency over the previous 3 months. Ultimately, based on their training levels, the runners were randomly assigned to the Elite Sodium Alginate Energy Gel group (ES) with 27 members, the Elite Traditional Energy Gel group (ET) with 27 members, the Advanced Sodium Alginate Energy Gel group (AS) with 19 members, and Advanced Traditional Energy Gel group (AT) with 20 members, ensuring comparable training backgrounds among participants. The runners utilized continuous glucose monitoring (CGM) devices for a week leading up to the race, regularly uploading their dietary and training logs. They were permitted to sample the energy gels prior to the event and were advised against a high-carbohydrate diet before the race. Due to various factors, including some participants being unable to finish the race due to health issues, CGM device malfunctions, and incomplete running data, 81 participants were ultimately included in the statistical analysis. The final sample size included in the statistical analysis (n = 81) was subjected to post hoc testing using G*Power software, yielding a power value of 0.82, which is consistent with the power values reported in previous studies (Sutehall et al., 2020; Inamura et al., 2024; Prins et al., 2025). Participant demographics are detailed in Table 1. A two-sample t-test indicated no significant differences in running volume over the past 3 months between the ES and ET groups (F = 0.0051, P = 0.84, η^2^p = 0.0001), or between the AS and AT groups (F = 0.0481, P = 0.83, η^2^p = 0.0013). Additionally, there were no significant differences in previous PB performances (F = 1.6115, P = 0.21, η^2^p = 0.03) (F = 1.321, P = 0.29, η^2^p = 0.031), suggesting comparable abilities among the runners in each group. No notable differences were found in age, BMI, height, weight, or baseline blood glucose levels across the groups. Race performance data were obtained from the 2024 Nanjing Marathon. This study received approval from the Human Experiment Ethics Committee of Nanjing Sport Institute (Approval No.: RT-2024–09), and all procedures adhered to the ethical standards set by the World Medical Association (Declaration of Helsinki). All participants provided written informed consent prior to the commencement of the study.

**TABLE 1:** Basic information of subjects.

Characteristics	ES (n = 22)	ET (n = 24)	P
Age (years)	38 ± 7	39 ± 8	0.86
BMI(body mass index)	21.63 ± 1.67	21.48 ± 1.03	0.93
Height (cm)	174.41 ± 3.88	174.28 ± 5.77	0.78
Weight (kg)	65.82 ± 5.87	65.32 ± 5.63	0.91
PB(personal best)	2:56:53 ± 0:06:51	3:03:03 ± 0:17:35	0.22
Running volume in recent three months (km)	900.10 ± 279.21	990.76 ± 307.27	0.79
Basal blood glucose (mmol/L)	4.956 ± 0.347	5.056 ± 0.563	0.48

	AS (n = 18)	AT (n = 17)	P
Age (years)	41 ± 7	45 ± 8	0.12
BMI	21.77 ± 1.44	22.14 ± 1.95	0.63
Height (cm)	173.83 ± 4.93	172.56 ± 4.97	0.72
Weight (kg)	65.82 ± 5.77	65.91 ± 6.30	0.45
Previous personal best	3:31:45 ± 0:19:22	3:39:10 ± 0:22:36	0.21
Running distance in recent three months (km)	709.97 ± 291.28	709.97 ± 291.28	0.32
Basal blood glucose (mmol/L)	4.954 ± 0.405	4.962 ± 0.484	0.96

### 2.2 Research methods

All participants in this study took part in the Nanjing Marathon on 17 November 2024, with an average temperature of 15 °C and a relative humidity of 76%, which are suitable climatic conditions for marathon running. The Nanjing Marathon is recognized as a Silver Label event by the International Association of Athletics Federations (IAAF). According to the recommendations of the American College of Sports Medicine (ACSM), athletes should consume 30–60 g of carbohydrates during endurance events lasting more than 1 hour. Based on this, this study developed personalized nutrition strategies for each participant. Carbohydrate intake followed a standard of 60 g per hour, with the total carbohydrate intake calculated based on their expected performance in the race. The specific energy replenishment plan is as follows: The Nanjing Marathon has set up a total of 19 supply stations (one every 2 km), with each station providing drinking water and sports drinks. This study requires runners to follow the principle of stopping at every station, alternating between sports drinks and drinking water. Each time they enter a sports drink supply station, runners are required to consume two-thirds of a cup (100 mL) of sports drink (the fluid intake meets the recommended replenishment guidelines). Based on the nutritional composition of the sports drink provided by the Nanjing Marathon, each intake of sports drink contains 6 g of carbohydrates. The sodium alginate energy gel used in the experiment was M-Action Fast-Endura^TM^ Energy Cruiser (Nutritional Dietary Supplement developed and produced by Shanghai M-Action Health Technology Co., Ltd.), containing 24.8 g of carbohydrates per serving, the traditional energy gel provided in this study was CPT PRO (Nutritional Dietary Supplement developed and produced by Beijing Competitor Sports Science Technology Joint Stock Co., Ltd.), and the carbohydrate content of a single energy gel was 28.92 g. We compared the other ingredients present in the two energy gels and found that none contained components that could potentially influence the research indicators. Energy gels are to be consumed before entering the drinking water supply stations. Calculation formula for energy gel replenishment frequency:

$$\text{Number of energy gel replenishments}=\frac{60g\times \text{Target}\;\text{finish}\;\text{time}-\text{serve}\;\text{refills}\;\text{of}\;\text{sports}\;\text{drinks}\left(g\right)\times \text{Sports}\;\text{drink}\;\text{refills}}{\text{Carbohydrate}\;\text{content}\;\text{of}\;\text{energy}\;\text{gel}\;\text{per}\;\text{single}\;\text{serving}\left(g\right)}$$

During the race, runners are allowed to consume bananas 2–3 times, with 1/3 of a banana each time, but they are not permitted to eat any other carbohydrate-containing foods besides bananas. If runners experience difficulty in consuming energy gels or mild gastrointestinal discomfort in the latter stages of the race, they are allowed to discontinue supplementation. Runners are required to finish their breakfast 2 hours before the start of the race, and they should not consume any carbohydrates from the time they finish breakfast until the race begins. Pre-race and in-race dietary and supplementation information will be recorded via a post-race questionnaire. Table 2 presents the energy supplementation plan of a runner in the ES group with a target time of 2 h and 33 min. The supplementation strategy for all participants was formulated following the same principle (60 g of carbohydrates per hour), with slight adjustments made to the specific timing of intake based on their target finish time and personal supplementation habits.

**TABLE 2:** Supply plan example–ES group runner, completion time: 02:33:00.

2024 Nanjing marathon personal energy supply plan				
Runner category	Elite runner	Supply group	Sodium alginate energy gel	
Name	**	Target time	02:33:00	
Station order	Station type	Kilometer	Supply requirement	Supply amount
1	Drink/Water	5	1 energy gel + water	2/3 paper cup
2	Drink/Water	7	Water	2/3 paper cup
3	Drink/Water	9	Water	2/3 paper cup
4	Drink/Water	11	Water	2/3 paper cup
5	Drink/Water	13	1 energy gel + water	2/3 paper cup
6	Drink/Water/Food	15	Water	2/3 paper cup
7	Drink/Water	17	Water	2/3 paper cup
8	Drink/Water	19	Water	2/3 paper cup
9	Drink/Water/Food	21	1 energy gel + water	2/3 paper cup
10	Drink/Water	23	Water	2/3 paper cup
11	Drink/Water	25	Water	2/3 paper cup
12	Drink/Water/Food	27	Water	2/3 paper cup
13	Drink/Water	29	1 energy gel + water	2/3 paper cup
14	Drink/Water/Food	31	Water	2/3 paper cup
15	Drink/Water/Food	33	Sports drink	2/3 paper cup
16	Drink/Water/Food	35	1 energy gel + water	2/3 paper cup
17	Drink/Water/Food	37	Water	2/3 paper cup
18	Drink/Water/Food	39	1 energy gel + water	2/3 paper cup
19	Drink/Water	41	Sports drink	2/3 paper cup

### 2.3 Data processing

#### 2.3.1 Running data collection and standardization

Athletes’ performance metrics were captured through the sports watches they utilized, with the data subsequently exported and stored via the associated application. The sports watches worn by runners are concentrated in brands such as Garmin, COROS, HUAWEI, and so on. To eliminate the individual differences in pace among runners of different levels and the potential measurement discrepancies of various sports watches, referring to the evaluation method of marathon pace fluctuation in previous studies (Knechtle et al., 2022), the formula used to normalize the runners’ pace per kilometer was as follows: Standardized pace (percentage change in pace) = 100% * (pace per kilometer - individual average pace throughout the race)/individual average pace throughout the race, with all percentages expressed in absolute values. To quantify the uncertainty of standardized metrics, we calculated the 95% confidence interval for the mean of each standardized metric using the t-distribution, with the standard error derived from the standardized data itself. The official race certificates served as the basis for the runners’ performance outcomes.

#### 2.3.2 Blood glucose data collection and standardization

The real-time blood glucose data of runners during the competition was obtained through the Sibionics Dynamic Blood Glucose Monitor, which can continuously monitor blood glucose data for 14 days. The blood glucose monitoring device was developed and manufactured by Shenzhen Sibionics Technology Co., Ltd. The device has received approval from the National Medical Products Administration in China (class III medical device) and has obtained international certification (compliance with the EU MDR medical device regulations). The Continuous Glucose Monitoring System measures the glucose concentration in the interstitial fluid beneath the skin through a subcutaneously implanted sensor. The test-retest reliability of the Continuous Glucose Monitoring (CGM) device, specifically the Silicon Sensing Dynamic Glucose Monitoring System, utilized in this study has been validated within the diabetic patient population (Yan et al., 2023). Participants were required to wear the device for 7 days prior to the competition, with the specific installation site being the back of the upper arm as specified in the product manual, to avoid any impact on the runners’ normal life and exercise. The sensor reads the glucose concentration in the interstitial fluid every 5 minutes, transmits the data via Bluetooth to the APP for real-time display and export processing. The sensor detects interstitial glucose concentrations every 5 minutes and transmits the data via Bluetooth to an application for real-time display. The average blood glucose value in the early morning 3 days prior to the competition will serve as the baseline. (Martone et al., 2024). To eliminate individual differences, the following formula is used to standardize the runners’ blood glucose data: Standardized blood glucose (%) = [(Average blood glucose per kilometer - Blood glucose baseline)/Blood glucose baseline] × 100%. The standardized glucose fluctuation range (maximum value - minimum value) is used to assess glucose variability, and the 95% confidence interval (t-distribution) of its mean is calculated based on the standardized data to measure statistical uncertainty.

### 2.4 Statistical methods

All results are presented as mean ± standard deviation (Migliorini et al., 2023). The key outcome metrics include the percentage change in pace per 10 km, the mean standardized blood glucose level per 10 km, and the fluctuation range of standardized blood glucose levels per 10 km. This study employs multifactorial repeated measures ANOVA to examine the effects of different fueling strategies on the pace and blood glucose data of runners at varying levels, with level (Group E runners and Group A runners), segment (10 km), and fueling type (Group S and Group T) as fixed effects. Multiple comparison analysis was conducted to assess differences in pace and blood glucose data among runners of different levels at various time points. One-way ANOVA was utilized to compare differences in each indicator among runners with different fueling strategies at the same time point. This study conducted a substantial number of multiple comparisons. To control the family-wise error rate, all post hoc pairwise comparison P-values were adjusted using the Bonferroni correction. After adjustment, a P-value of less than 0.05 was deemed statistically significant. Effect size was expressed using η^2^p, and all data were statistically analyzed using SAS JMP Pro 17.0.0.

## 3 Results

### 3.1 Statistical analysis of race completion

Table 3 presents the supply status and race completion times for participants across different groups. The analysis revealed no significant differences in carbohydrate consumption from energy gels or sports beverages among the groups (F = 2.1204, P = 0.09, η^2^p = 0.064) (F = 2.0343, P = 0.99, η^2^p = 0.043), confirming the effectiveness of the study’s design and the comparability of the groups. A two-sample T-test was performed on the race completion times, indicating that the ES group’s average time was superior to that of the ET group, and the AS group’s average time outperformed the AT group. However, no significant differences were found in completion times across the groups (F = 1.1533, P = 0.29, η^2^p = 0.024) (F = 1.0543, P = 0.31, η^2^p = 0.031). Furthermore, the incidence of gastrointestinal discomfort among runners in the ES group was comparable to that in the ET group, whereas the AS group reported a lower incidence than the AT group.

**TABLE 3:** Participants’ in-race supplementation and race completion results.

Race completion data	ES (n = 22)	ET (n = 24)	P
Carbohydrate from energy gels (g)	122.46 ± 28.94	125.32 ± 27.85	0.94
Carbohydrate from sports drinks (g)	41.45 ± 13.59	45 ± 6.85	0.26
Total carbohydrate intake (g)	163.91 ± 6.29	170.32 ± 5.64	0.99
Race finish Time (hh:mm:ss)	2:59:50 ± 0:09:53	3:06:22 ± 0:27:36	0.29
Average Pace(min/km)	0:04:12 ± 0:00:19	0:04:24 ± 0:00:45	0.21

	AS (n = 18)	AT (n = 17)	P
Carbohydrate from energy gels (g)	141.91 ± 20.49	152.31 ± 20.35	0.16
Carbohydrate from sports drinks (g)	44 ± 11.82	46.4 ± 5.30	0.47
Total carbohydrate intake (g)	185.91 ± 5.28	198.71 ± 18.57	0.09
Race finish Time (hh:mm:ss)	3:34:39 ± 0:23:53	3:43:31 ± 0:27:26	0.31
Average pace (hh:mm:ss/km)	0:05:03 ± 0:00:37	0:05:14 ± 0:00:44	0.38

Total Carbohydrate Intake = Energy gel + sports drink + banana. All intergroup comparisons showed P values >0.05. Pace is measured in hh:mm:ss per kilometer (hh:mm:ss/km), indicating the time taken to cover 1 km.

**FIGURE 1:**
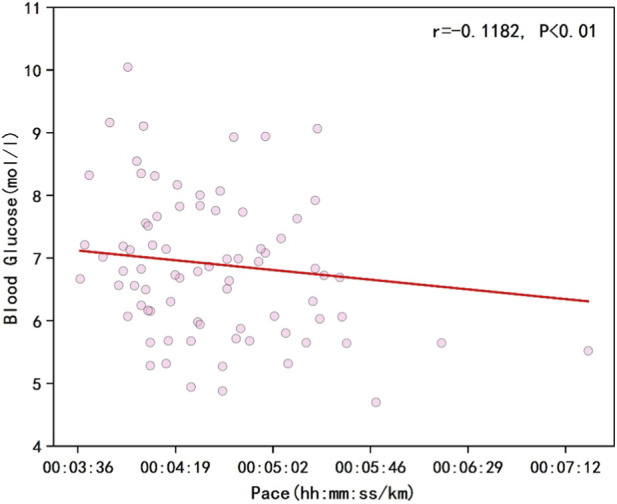
The correlation between pace and blood glucose levels in all runners.

**FIGURE 2:**
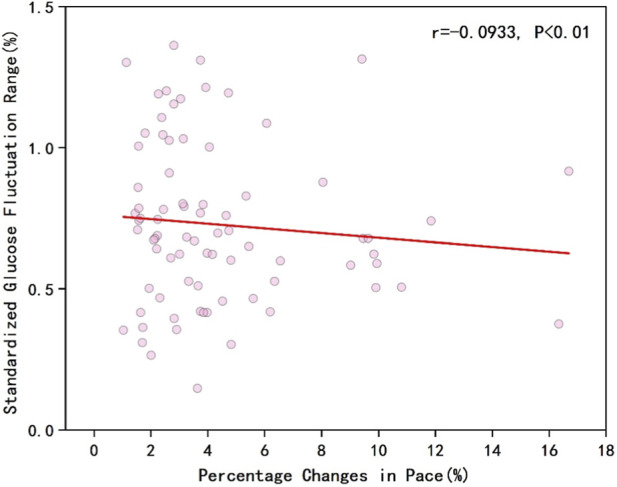
The correlation between percentage changes in pace and standardized glucose fluctuation range in all runners.

**FIGURE 3:**
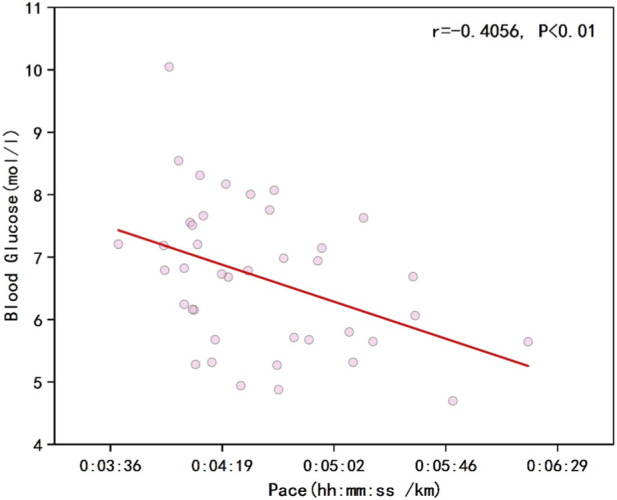
The correlation between Runner’s pace and blood glucose in the sodium alginate group.

**FIGURE 4:**
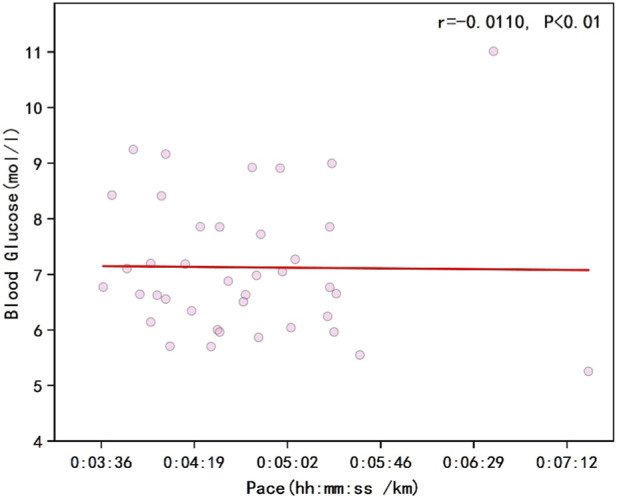
The correlation between Runner’s pace and blood glucose in the traditional energy gel group.

As shown in Figure 1, the results indicated a negative correlation between runners’ overall pace and blood glucose levels (r = −0.1182, P < 0.01). Figure 2 illustrates that the percentage changes in overall runner pace were negatively correlated with the standardized glucose fluctuation range (r = −0.0933, P < 0.01). Figure 3 demonstrates a significant negative correlation between running pace and blood glucose levels in the sodium alginate energy gel group (r = −0.4056, P < 0.01), whereas Figure 4 shows that the correlation in the traditional energy gel group was relatively weak (r = −0.0110, P < 0.01).

### 3.2 Analysis of percentage change in pace per 10 km segment

**FIGURE 5:**
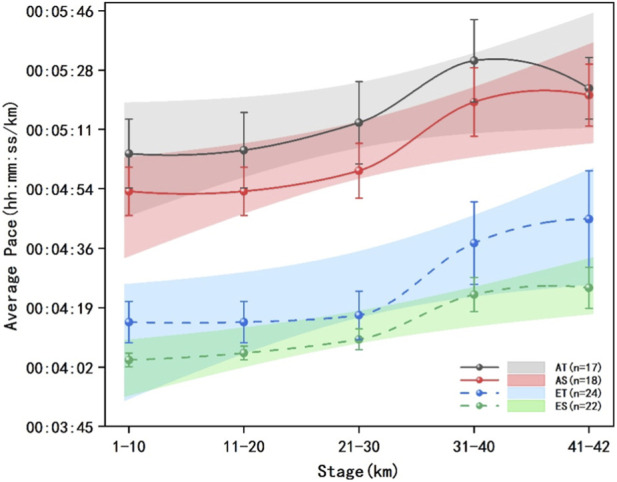
Mean pace of each group across 5 stages. The data presented comprises solid and dashed lines that represent five race stages for different groups of runners across various performance levels, the shaded areas denote the 95% confidence intervals, the error bars represent the standard error, advanced traditional energy gel (AT): black solid line and shaded area, advanced sodium alginate energy gel (AS): red solid line and shaded area, elite traditional energy gel (ET): blue dashed line and shaded area, elite sodium alginate energy gel (ES): green dashed line and shaded area.

**FIGURE 6:**
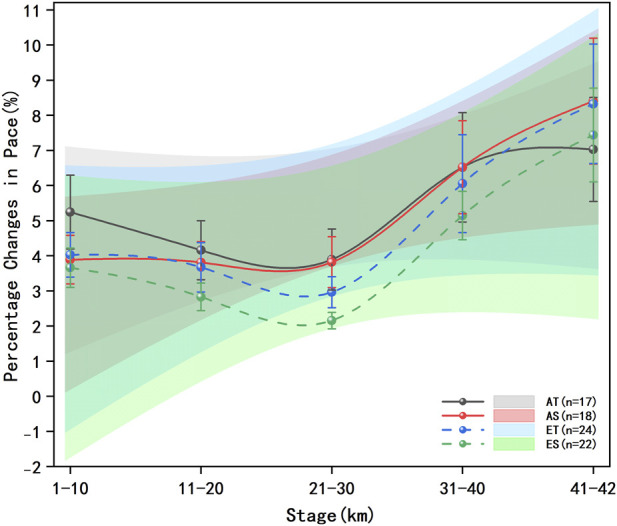
Percentage changes in pace across five segments for each group. The data presented comprises solid and dashed lines that represent five race stages for different groups of runners across various performance levels, the shaded areas denote the 95% confidence intervals, the error bars represent the standard error, advanced traditional energy gel (AT): black solid line and shaded area, advanced sodium alginate energy gel (AS): red solid line and shaded area, elite traditional energy gel (ET): blue dashed line and shaded area, elite sodium alginate energy gel (ES): green dashed line and shaded area.

The average pace per 10 km and the percentage changes in pace per 10 km for each group of runners are presented in Figures 5, 6. The results of multivariate analysis of variance showed that the sodium alginate group demonstrated higher but not statistically significant average pace at the same time points across different distance segments compared to the traditional energy gel group (F = 0.0245, P = 0.88, η^2^p = 0.0003). The percentage changes in pace among the groups of runners had statistically significant differences across the race segments (F = 0.9437, P < 0.01, η^2^p = 0.4855), indicating that the percentage changes in pace were not entirely consistent across different segments of the race for each group of runners. The percentage changes in pace by segments showed significant differences in terms of energy gel type (F = 0.0035, P < 0.01, η^2^p = 0.0035), runner level (F = 0.0096, P < 0.01, η^2^P < 0.01), and their interaction (F = 0.0023, P < 0.01, η^2^p = 0.0023).

The percentage change in pace for the AT group was significantly higher than that of the AS group in the 1–10 km segment (F = 0.0397, P < 0.01, η^2^p = 0.0382) and the 11–20 km segment (F = 0.0039, P = 0.02, η^2^p = 0.0039), while it was significantly lower in the 41–42 km segment (F = 0.0108, P = 0.01, η^2^p = 0.0107). The ET group showed significantly higher percentage changes in pace across all five race segments compared to the ES group (F = 0.0042, P < 0.01, η^2^p = 0.0042) (F = 0.0238, P < 0.01, η^2^p = 0.0232) (F = 0.0559, P < 0.01, η^2^p = 0.0529) (F = 0.0074, P < 0.01, η^2^p = 0.0074) (F = 0.0037, P < 0.01, η^2^p = 0.0037).

Multiple comparisons indicated that the percentage change in pace for runners in the AT group was significantly lower in the 11–20 km segment compared to the 1–10 km segment (F = 0.3528, p < 0.01, η^2^p = 0.2608), yet significantly higher than in the 21–30 km segment (F = 0.0248, p < 0.01, η^2^p = 0.0242). Furthermore, the percentage change in pace in the 31–40 km segment was significantly higher than that in the 21–30 km segment (F = 0.4881, p < 0.01, η^2^p = 0.3280), but significantly lower than in the 41–42 km segment (F = 0.0186, p < 0.01, η^2^p = 0.0183).

For the AS group, the percentage change in pace was significantly higher in the 31–40 km segment compared to the 21–30 km segment (F = 0.2601, p < 0.01, η^2^p = 0.2065), while it was significantly lower than that in the 41–42 km segment (F = 0.2601, p < 0.01, η^2^p = 0.2065).

In the ET group, multiple comparisons revealed that the percentage change in pace for the 11–20 km segment was significantly lower than in the 1–10 km segment (F = 0.0808, p < 0.01, η^2^p = 0.0747), yet significantly higher than in the 21–30 km segment (F = 0.1473, p < 0.01, η^2^p = 0.1284). The percentage change in pace for the 31–40 km segment was significantly higher than that for the 21–30 km segment (F = 0.3821, p < 0.01, η^2^p = 0.2765), and significantly lower than that for the 41–42 km segment (F = 0.1300, p < 0.01, η^2^p = 0.1151).

Lastly, in the ES group, the percentage change in pace for the 11–20 km segment was significantly lower than for the 1–10 km segment (F = 0.2014, p < 0.01, η^2^p = 0.1676), yet significantly higher than for the 21–30 km segment (F = 0.3076, p < 0.01, η^2^p = 0.2352). The percentage change in pace for the 31–40 km segment was significantly higher than that of the 21–30 km segment (F = 1.1771, p < 0.01, η^2^p = 0.5407), but significantly lower than that of the 41–42 km segment (F = 0.1487, p < 0.01, η^2^p = 0.1295).

### 3.3 10 km segmented standardized blood glucose analysis

**FIGURE 7:**
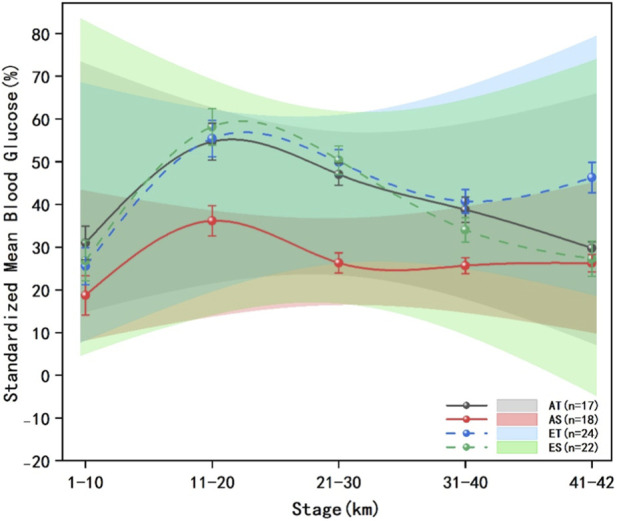
Standardized mean blood glucose levels across 5 stages in each group. The data presented comprises solid and dashed lines that represent five race stages for different groups of runners across various performance levels, the shaded areas denote the 95% confidence intervals, the error bars represent the standard error, advanced traditional energy gel (AT): black solid line and shaded area, advanced sodium alginate energy gel (AS): red solid line and shaded area, elite traditional energy gel (ET): blue dashed line and shaded area, elite sodium alginate energy gel (ES): green dashed line and shaded area.

**FIGURE 8:**
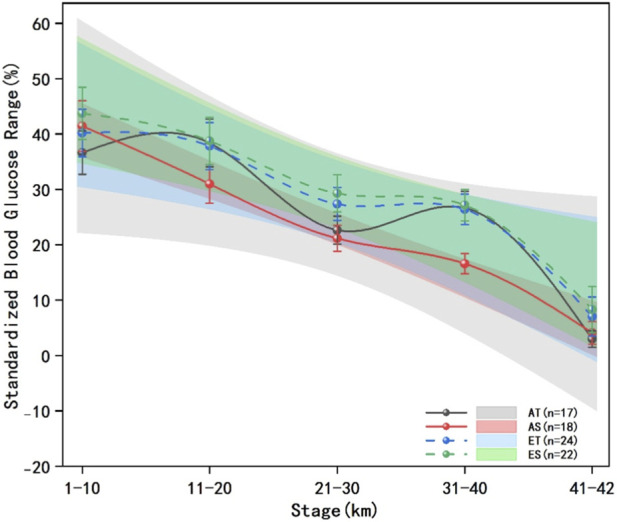
Standardized blood glucose fluctuation range across five stages in each group. The data presented comprises solid and dashed lines that represent five race stages for different groups of runners across various performance levels, the shaded areas denote the 95% confidence intervals, the error bars represent the standard error, advanced traditional energy gel (AT): black solid line and shaded area, advanced sodium alginate energy gel (AS): red solid line and shaded area, elite traditional energy gel (ET): blue dashed line and shaded area, elite sodium alginate energy gel (ES): green dashed line and shaded area.

The blood glucose data of runners from each group at every 10 km are presented in Figures 7, 8. The results of multivariate analysis of variance showed that the standardized mean blood glucose levels of runners in each group exhibited statistically significant differences across the race segments (F = 1.1879, P < 0.01, η^2^p = 0.5429), indicating that the blood glucose levels of runners in each group were not all equal at different time points during the race. The standardized mean blood glucose levels of runners in each group at 10-km segments showed significant differences in terms of energy gel type (F = 0.0376, P < 0.01, η^2^p = 0.0362), runner level (F = 0.0303, P < 0.01, η^2^p = 0.0294), and their interaction (F = 0.0104, P < 0.01, η^2^p = 0.0103).

Multiple comparisons revealed that the standardized glucose fluctuation range in the AT group was significantly lower than that in the AS group at 1–10 km (F = 0.0245, P < 0.01, η^2^p = 0.0240) and 41–42 km (F = 0.0320, P < 0.01, η^2^p = 0.0310), while it was significantly higher in the AT group compared to the AS group at 11–20 km (F = 0.0293, P < 0.01, η^2^p = 0.0285) and 31–40 km (F = 0.1392, P < 0.01, η^2^p = 0.1225). The standardized blood glucose fluctuation range of ET group was significantly lower than that of ES group at 1–10 km (F = 0.0088, P < 0.01, η^2^p = 0.0086) and 41–42 km (F = 0.0088, P < 0.01 η^2^p = 0.0087).

The standardized mean blood glucose level in the AT group was significantly higher in the 11–20 km segment compared to the 1–10 km segment (F = 0.8853, P < 0.01, η^2^p = 0.4696) and the 21–30 km segment (F = 0.1794, P < 0.01, η^2^p = 0.1521). The standardized mean blood glucose levels in the 31–40 km (F = 0.1588, P < 0.01, η^2^p = 0.1370) and 41–42 km segments (F = 0.3134, P < 0.01, η^2^p = 0.2389) were significantly lower than those in their preceding segments.

The standardized blood glucose mean of the AS group was significantly higher in the 11–20 km segment compared to the 1–10 km segment (F = 1.1176, P < 0.01, η^2^p = 0.5278) and the 21–30 km segment (F = 0.8262, P < 0.01, η^2^p = 0.4524). Additionally, the 31–40 km segment exhibited significantly lower blood glucose levels than the 21–30 km segment (F = 0.0063, P = 0.0293, η^2^p = 0.0063).

The standardized mean blood glucose level in the ET group was significantly higher in the 11–20 km segment compared to both the 1–10 km segment (F = 1.4543, P < 0.01, η^2^p = 0.5929) and the 21–30 km segment (F = 0.0424, P < 0.01, η^2^p = 0.0406). Conversely, the standardized mean blood glucose level in the 31–40 km segment was significantly lower than that in the 21–30 km segment (F = 0.2326, P < 0.01, η^2^p = 0.1887) and the 41–42 km segment (F = 0.1126, P < 0.01, η^2^p = 0.1012).

In the ES group, the standardized mean blood glucose level was also significantly higher in the 11–20 km segment compared to the 1–10 km segment (F = 1.5209, P < 0.01, η^2^p = 0.6033) and the 21–30 km segment (F = 0.1347, P < 0.01, η^2^p = 0.1187). Furthermore, the standardized mean blood glucose level in the 31–40 km segment was significantly lower than that in the 21–30 km segment (F = 0.6118, P < 0.01, η^2^p = 0.3796) but significantly higher than that in the 41–42 km segment (F = 0.1189, P < 0.01, η^2^p = 0.1063).

## 4 Discussion

In endurance competitions like marathons, the proper replenishment of energy is vital for athletes’ success. Carbohydrate oxidation is the main energy source during extended physical activity for endurance competitors (Wardenaar et al., 2018), and consuming carbohydrates while racing has been shown to enhance performance and mitigate fatigue (Costa et al., 2016; Inamura et al., 2024). Nonetheless, athletes exhibit considerable differences in how they absorb and tolerate carbohydrates, which can be affected by their individual gastrointestinal responses and the osmolarity of the supplements they consume (Ishihara et al., 2020; Lavoué et al., 2020). Traditional energy gels generally have high osmolarity (Zhang et al., 2015), and while hypertonic gels can deliver energy quickly, they may also draw water into the intestines, leading to discomfort such as bloating, nausea, and diarrhea (Miyashita et al., 2019; Lavoué et al., 2020). This highlights a major limitation of current energy gels: although they offer quick energy and convenience, they do not provide the adaptable energy release that better suits the body’s digestive and absorption requirements during physical exertion. To tackle this issue, ongoing advancements in carbohydrate supplementation are necessary. This study research aims to utilize a novel encapsulation technology-based carbohydrate (sodium alginate energy gel) as an endurance exercise energy supplement, and to evaluate its effects on blood sugar stability and athletic performance in marathon runners compared to traditional energy gels. Sodium alginate, a natural polysaccharide, consists of β-D-mannuronic acid (M) and α-L-guluronic acid (G) units. This unique structure enables the formation of a three-dimensional network gel through ionic cross-linking in the acidic environment of the stomach following ingestion (Sutehall et al., 2022b). This gel effectively encapsulates high-sugar and high-energy substances, alleviating gastric discomfort and accelerating gastric emptying, thereby reducing the residence time of nutrients in the stomach (Zhang et al., 2015; Sutehall et al., 2020). Upon entering the small intestine, the sodium alginate gel expands as pH increases, gradually releasing and absorbing the encapsulated high-sugar and high-energy nutrients (Sutehall et al., 2022b). This study validated the sustained-release mechanism in real marathon scenarios, with results consistent with laboratory research. Consequently, sodium alginate gel plays a vital role in providing high-sugar and high-energy nutrients more consistently, which is crucial for endurance performance, particularly in scenarios that demand prolonged and sustained energy supply (Aliyu et al., 2023).

In actual marathon events, the way energy sources are utilized varies significantly throughout different phases of the race (MoTrPAC Study Group et al., 2024; Valder et al., 2024), contrasting sharply with the controlled steady-state conditions found in lab settings. Consequently, this research focuses on a real marathon, breaking down the full 42 km distance into five distinct segments (1–10 km, 11–20 km, 21–30 km, 31–40 km, 41–42 km). The results revealed that all groups of runners exhibited a consistent pattern in pace changes, showing a gradual decline in pace variation during the 1–30 km segments (p < 0.05), followed by a notable and steady rise in pace variation during the 21–42 km segments (p < 0.05). Blood glucose levels for all groups initially rose before falling, with the average levels during the 11–20 km segment being significantly elevated compared to other segments (P < 0.01). This observation suggests that runners of similar abilities, irrespective of the energy supplements they use, display comparable shifts in metabolic traits and energy requirements.

In prolonged endurance sports such as marathons, the close relationship between pacing and blood glucose has been extensively studied (Flockhart and Larsen, 2024). However, the stability of blood glucose and its impact on athletic performance still hold many unresolved mysteries. Research shows that elite runners often demonstrate high pacing stability during competitions, yet their blood glucose fluctuations are more pronounced, suggesting that blood glucose stability may not be the sole determinant of pacing (Suzuki et al., 2015; Inamura et al., 2024). Furthermore, the differences in energy substrate utilization between advanced runners and elite runners are also worth in-depth exploration. Advanced runners may rely more on fat as an energy source, whereas elite runners tend to utilize carbohydrates more efficiently, especially during high-intensity exercise (Maunder et al., 2018; Inamura et al., 2024). This disparity may be attributed to variations in training levels, metabolic adaptability, and individual physiological characteristics. Furthermore, in this study, the proportion of runners experiencing gastrointestinal discomfort in the ES group (4.55%) was comparable to that in the ET group (4.17%), whereas the proportion in the AS group (0%) was significantly lower than that in the AT group (5.88%). This further underscores the differences in gastrointestinal tolerance among runners of varying levels. Therefore, runners of varying levels may exhibit different effects after consuming energy gels at different stages of the race.

This study found that the standardized blood glucose fluctuation range of sodium alginate energy gel group significantly increased during the 1–10 km and 41–42 km segments. In the 11–20 km and 31–40 km segments, runners in the advanced sodium alginate energy gel group demonstrated better blood glucose stability. This stability may be attributed to the unique properties of sodium alginate and its absorption dynamics within the digestive system. The gel form of sodium alginate may enhance gastric emptying, which could facilitate a quicker energy release. Additionally, the simple sugars (glucose and fructose) present in these gels are absorbed swiftly by the small intestine via SGLT1 and GLUT5 transporters, leading to a rapid increase in blood sugar levels (Cook et al., 2023; Liao et al., 2024). However, the increase remains within the normal range of blood sugar fluctuations, effectively supporting exercise performance, as evidenced by the significantly faster pace of this group compared to the traditional energy group. Additionally, the factors influencing blood sugar fluctuations are not limited to the type of energy substrate and absorption rate but also include exercise intensity, individual metabolic state, and environmental conditions. Studies have shown that during the initial and final stages of exercise, athletes’ metabolic demands significantly increase compared to periods of inactivity or before sprinting (Inamura et al., 2024), prompting the liver to release more glucose into the bloodstream, which results in elevated blood sugar levels (Suzuki et al., 2015; Inamura et al., 2024). This observation aligns with existing literature on the intricate relationship between energy supplementation and glucose regulation (Santos et al., 2022). During the mid-phase of a marathon, runners’ energy demands are high but tend to stabilize. Sodium alginate energy gels may more effectively sustain blood glucose levels and reduce fluctuations (Bizjak et al., 2022). In the latter stages of the marathon, the energy derived from these gels may be utilized more efficiently by runners, helping to sustain their speed, which is vital for maintaining performance in the final stretch. Consequently, despite experiencing greater fluctuations in blood glucose levels, the sodium alginate group still outperformed the traditional energy gel group in terms of pace. These findings further support the mechanism by which sodium alginate gels modulate glucose and fructose release rates through their pH-sensitive swelling and shrinking properties (Zhao et al., 2024). The observation in this study that sodium alginate gel promotes the stabilization of blood glucose levels bears similarity to the “gradient regulation” principle of alginate-chitosan composite hydrogel controlling S1P release proposed in previous studies (Williams et al., 2017). However, this study is the first to apply it in a real exercise scenario, by regulating the rate of carbohydrate digestion and absorption (Huang et al., 2023), thereby regulating blood glucose homeostasis and optimizing energy metabolism patterns (König et al., 2016), to achieve enhanced stability in exercise performance. The blood glucose fluctuations observed in the real-world application of sodium alginate energy gels may have a dual impact on athletic performance: on one hand, the rapidly absorbed monosaccharides can quickly provide energy, delaying fatigue with a fast onset; on the other hand, the sharp fluctuations in blood glucose may lead to instability in energy supply, potentially affecting pacing and overall performance (Kazemi et al., 2023). However, this study found that in terms of speed control, elite runners using sodium alginate energy gels showed significantly lower percentage changes in speed across all five race segments compared to the traditional energy gel group (p < 0.01), indicating that sodium alginate energy gels may have a significant advantage in maintaining long-term pacing stability for runners. Additionally, the gel properties of sodium alginate seem to improve gastrointestinal tolerance, reduce gastrointestinal discomfort, and further enhance the athletic performance of runners. The performance of sodium alginate energy gel in pacing among elite runners is particularly notable, which may also be related to the metabolic adaptability and training level of these runners. Studies suggest that endurance athletes with extensive training have superior blood glucose regulation, allowing for more efficient carbohydrate utilization (Inamura et al., 2024). The gastrointestinal digestion characteristics of sodium alginate energy gel, combined with this metabolic adaptability, may further optimize the regulation of blood glucose. Advanced runners experienced a significant reduction in the percentage of speed variation (p < 0.05) after using sodium alginate energy gel in the 1–10 km and 11–20 km segments, which further validates the universal applicability of sodium alginate energy gel among runners of different levels.

Previous studies have suggested that maintaining blood glucose stability during endurance exercise significantly impacts athletes’ pacing stability and race performance (Suzuki et al., 2015). When muscle glycogen reserves are depleted, glucose in the bloodstream becomes the primary energy source for both the brain and muscle tissues. Under such conditions, marked hypoglycemia or an inability to maintain glucose availability may lead to fatigue and the classic phenomenon of ‘glycogen depletion’ (Chycki et al., 2018). However, our correlation analyses challenge the simplified interpretation that ‘greater glucose stability necessarily leads to more stable pacing.’ This study identified a negative correlation between pacing and blood glucose levels among runners at varying paces. Specifically, as the runners’ pace increased, their blood glucose levels also rose. This phenomenon may be attributed to enhanced glycogenolysis and gluconeogenesis during exercise, particularly in high-intensity workouts, where the body preferentially utilizes blood glucose as an energy source, leading to elevated blood glucose levels (Suzuki et al., 2015). Additionally, changes in insulin sensitivity during exercise may further influence blood glucose dynamics. Research indicates that insulin secretion and action may be suppressed during physical activity, exacerbating the rise in blood glucose levels (Ben Brahim et al., 2015). Importantly, these observations suggest that blood glucose concentration during racing reflects not only ‘fuel sufficiency’ but also the integrated response of exercise intensity, hepatic glucose production, and hormonal regulation. Moreover, we identified a seemingly counterintuitive relationship between pacing stability and the amplitude of blood glucose fluctuations. Traditional views generally consider blood glucose stability as the primary determinant of endurance exercise performance (Inamura et al., 2024). However, the statistical results of this study indicate a negative correlation between the percentage change in runners’ pacing and blood glucose fluctuations. Specifically, as pacing stability increases, the amplitude of blood glucose fluctuations also rises. A plausible explanation for this phenomenon is that pacing stability may reflect superior metabolic flexibility and regulatory responsiveness, rather than simply minimizing blood glucose fluctuations. As exercise continues, the balance between hepatic glucose production, muscle glycogen utilization, and fat oxidation is constantly readjusting. These changes in performance can result in fluctuations of blood glucose concentration even though performance does not have significant changes (Suzuki et al., 2015). Moreover, the secretion of hormones like adrenaline, cortisol, and glucagon during exercise may change the dynamic equilibrium of blood glucose concentration significantly. These hormones cause fluctuation of blood glucose levels that worsen under stable pacing conditions. In such cases, the metabolic regulatory mechanisms become more sensitive within the body, increasing the variation amplitude of blood glucose. Athletes capable of sustaining a consistent pace can potentially endure and absorb these fluctuations without an adverse impact on performance, given that glucose concentrations are maintained within a physiologically safe threshold (Suzuki et al., 2015). Overall, our data support the notion that it is not the glucose fluctuations that are performance limiting; rather, the outcome of pacing is shaped by multiple interacting determinants. It is worth noting that the relationship between glucose level and running pace was more evidence in the sodium alginate energy gel group. There was no association observed in runners consuming standard energy gels. This difference would be due to the differing gastrointestinal and absorption characteristics of the two formulations. The kinetics of carbohydrate availability may alter the intensity–glucose relationship during exercise, but this should not be misinterpreted as a direct ergogenic effect of high glucose concentrations. Thus, this interpretation may also help explain contradictions in the literature on carbohydrate availability and performance. Research studies that utilize a prolonged exercise followed by a performance test and the benefits of carbohydrate provision that commonly occur. Likely due to the fact that with prolonged exercise, substrate availability at late stages and central and peripheral fatigue become increasingly dominant (Kozlowski et al., 2021). In contrast, studies that use short-term time trials or experimental designs that increase blood glucose availability without activating the oral-gastrointestinal pathway and perceptual pathway are less likely to detect performance improvements, despite having elevated blood glucose (Carter et al., 2004). Therefore, the association between exercise performance and blood glucose levels may be context-dependent, varying with factors such as exercise duration, intensity distribution, carbohydrate delivery route, and the relative contributions of central versus peripheral fatigue mechanisms. In addition, endurance performance is influenced by a variety of factors. For instance, oxidative stress, which occurs when intense exercise leads to substantial free radical production that attacks tissues and contributes to functional decline and exercise-induced fatigue (Goulart et al., 2020). Inflammatory response and organ damage in which high-intensity exercise produces damage to muscles, kidneys, intestines, and other organs giving rise to systemic inflammation have been recognized as relevant influencing factors (Tominaga et al., 2021). Moreover, adaptation in skeletal muscle, cardiovascular function, psychological state, and recovery capacity also play an important role in physical fitness. Although the present study did not assess these mechanisms directly, they may serve as a benchmark for future studies that aim to further examine the regulatory mechanisms regulating endurance performance.

This research found no significant differences in the finishing times between runners using sodium alginate energy gels and those using traditional energy gels (p > 0.05). While the study highlighted the benefits of sodium alginate gels in terms of speed and blood sugar stability, these advantages did not translate into improved overall performance, likely due to several factors. One major reason could be the individual variability among athletes, which affects metabolic rates, gastrointestinal tolerance, and the efficiency of energy gel absorption (Özbay et al., 2024). Additionally, the energy demands fluctuate throughout a marathon, and the benefits of sodium alginate gels may not be applicable across all race segments. Future studies might explore the specific characteristics of different phases of a race. Furthermore, athletes’ pacing strategies and how they distribute their energy during the event could also play a role in their performance outcomes. Therefore, although sodium alginate energy gel can theoretically provide a more stable energy supply, its actual effectiveness may be influenced by a variety of complex factors. The advantages of sodium alginate energy gels in enhancing metabolic stability during critical race segments, as identified in this study, provide a valuable reference for formulating more personalized fueling strategies. These gels may serve as an effective alternative or supplementary option to traditional hypertonic energy gels, enabling runners—particularly elite athletes with higher metabolic efficiency—to better implement pre-race strategies, maintain neuromuscular recruitment efficiency, and mitigate fatigue resulting from abnormal blood sugar fluctuations (Pomportes et al., 2016). Ultimately, this translates into improved pace stability. Although the differences in average performance did not reach statistically significant levels, for athletes striving to maximize their performance, the enhancement in pace stability is a fundamental aspect of achieving excellence, and its importance should not be underestimated.

In summary, blood glucose levels and its stability during prolonged endurance exercise is a complex and multifaceted issue. This study demonstrates the application effects of sodium alginate energy gel in exercise scenarios, complementing the findings of Sutehall et al. (2022b) in resting states. The sodium alginate energy gel used in this study breaks through the formulation limitations of traditional energy gels: the pH responsiveness of sodium alginate constructs a delivery system (Wang et al., 2024). This feature allows for dynamic regulation, minimizing gastric discomfort while facilitating sustained absorption in the intestines (Wang et al., 2024). Compared to existing studies, the breakthrough finding of this research lies in revealing the regulatory effect of alginate energy gels on the pace stability in the first half of a marathon and mid-race blood glucose levels among runners of different levels, which provides new data for studying the relationship between blood glucose and athletic performance (Huang et al., 2023; Zhao et al., 2024). By employing continuous glucose monitoring (CGM) to track blood sugar fluctuations in an actual marathon environment, this research effectively links laboratory findings with real-world applications (Baur and Saunders, 2021). However, the differences in energy substrate utilization between elite and amateur runners, as well as the impact of sodium alginate on blood glucose fluctuations and its adaptability to different athletic populations, remain important directions for future research. By integrating advanced monitoring technologies and personalized nutrition strategies, we are poised to provide athletes with more scientific guidance, thereby enhancing their athletic performance and competitive results.

This study encountered several limitations, the most significant of which was participant attrition, as only 81 out of the initial 93 participants completed the race and provided complete data. The energy gels used in this study were commercial products, allowing participants to easily recognize their type through the packaging. Consequently, implementing a single-blind experimental design was not feasible. However, to maintain experimental control, participants were randomly grouped based on factors such as running volume, age, and skill level. Continuous glucose monitoring (CGM) was employed to track interstitial glucose levels, which typically lag behind blood glucose readings by 5–10 min, potentially leading to an underestimation of glucose fluctuation amplitudes. Variations in the brands of sports watches used by runners may influence data collection. Nonetheless, we mitigated errors through data smoothing and standardization. All data were collected from participants of the 2024 Nanjing Marathon, and the generalizability of the research findings may be influenced by climatic conditions and course characteristics. The conclusions need validation across various geographical environments and event types. Additionally, all participants were male, and no age-based grouping was implemented. The study did not consider potential physiological and metabolic differences that may exist between runners of different genders. Future research could enhance the robustness and generalizability of findings by utilizing mixed-gender and age-diverse samples, expanding sample sizes, and exploring various environmental conditions.

## 5 Conclusion

During the competition, the use of various energy gels did not lead to distinct effects on overall performance. Both types of energy gels effectively maintained endurance sports performance. However, the intake of sodium alginate energy gel significantly optimized blood sugar stability during the mid-section of the race for advanced runners and demonstrated improved pacing stability throughout the entire race for elite runners, as well as in the first half for advanced runners. This indicates that it contributes to stabilizing blood sugar levels and enhancing pacing stability in specific segments of the race. For elite runners, sodium alginate energy gels may be more suitable in scenarios where maintaining a stable pace throughout the race is crucial. Advanced runners are advised to prioritize the use of sodium alginate energy gels during the mid-race phase to effectively mitigate blood sugar fluctuations. Additionally, runners should assess their individual gastrointestinal tolerance and gradually adapt to the use of these new energy gels. Given the limitations regarding carbohydrate supplement dosages, further research is warranted to explore various types and dosages of energy gels supply plan to the characteristics of different race segments and individual tolerances. This will facilitate the optimization of sodium alginate technology and the development of more effective and scientifically grounded marathon race supplementation strategies.

## Data Availability

The original contributions presented in the study are included in the article/supplementary material, further inquiries can be directed to the corresponding authors.
